# Autonomous Microsystems for Downhole Applications: Design Challenges, Current State, and Initial Test Results

**DOI:** 10.3390/s17102190

**Published:** 2017-09-23

**Authors:** Myungjoon Choi, Yu Sui, In Hee Lee, Ryan Meredith, Yushu Ma, Gyouho Kim, David Blaauw, Yogesh B. Gianchandani, Tao Li

**Affiliations:** Center for Wireless Integrated MicroSensing and Systems (WIMS^2^), University of Michigan, Ann Arbor, MI 48109, USA; myungjun@umich.edu (M.C.); sui@umich.edu (Y.S.); inhee@umich.edu (I.H.L.); remered@umich.edu (R.M.); yushuma@umich.edu (Y.M.); gyouhokim@umich.edu (G.K.); blaauw@umich.edu (D.B.); yogesh@umich.edu (Y.B.G.)

**Keywords:** microsensors, pressure, temperature, encapsulation

## Abstract

This paper describes two platforms for autonomous sensing microsystems that are intended for deployment in chemically corrosive environments at elevated temperatures and pressures. Following the deployment period, the microsystems are retrieved, recharged, and interrogated wirelessly at close proximity. The first platform is the Michigan Micro Mote for High Temperature (M^3^HT), a chip stack 2.9 × 1.1 × 1.5 mm^3^ in size. It uses RF communications to support pre-deployment and post-retrieval functions, and it uses customized electronics to achieve ultralow power consumption, permitting the use of a chip-scale battery. The second platform is the Environmental Logging Microsystem (ELM). This system, which is 6.5 × 6.3 × 4.5 mm^3^ in size, uses the smallest suitable off-the-shelf electronic and battery components that are compatible with assembly on a flexible printed circuit board. Data are stored in non-volatile memory, permitting retrieval even after total power loss. Pre-deployment and post-retrieval functions are supported by optical communication. Two types of encapsulation methods are used to withstand high pressure and corrosive environments: an epoxy filled volume is used for the M^3^HT, and a hollow stainless-steel shell with a sapphire lid is used for both the M^3^HT and ELM. The encapsulated systems were successfully tested at temperature and pressure reaching 150 °C and 10,000 psi, in environments of concentrated brine, oil, and cement slurry. At elevated temperatures, the limited lifetimes of available batteries constrain the active deployment period to several hours.

## 1. Introduction

Downhole environmental monitoring is routinely required and practiced by the petroleum industry. Knowledge of geophysical conditions, such as temperature, pressure, and chemical concentrations in oil wells, hydraulic fractures, and reservoirs, can guide decisions governing operational efficiency and safety in oil exploration and production [1,2,3,4]. Conventional wireline logging methods use tethered instruments that sense local conditions when lowered into the wellbore [1]. In contrast, cross-well imaging methods use instruments within wellbores to generate and sense seismic or electromagnetic waves that propagate between wellbores; this data can be used to generate formation maps [5]. These techniques mainly provide aggregate information with limited spatial resolution.

**Figure 1:**
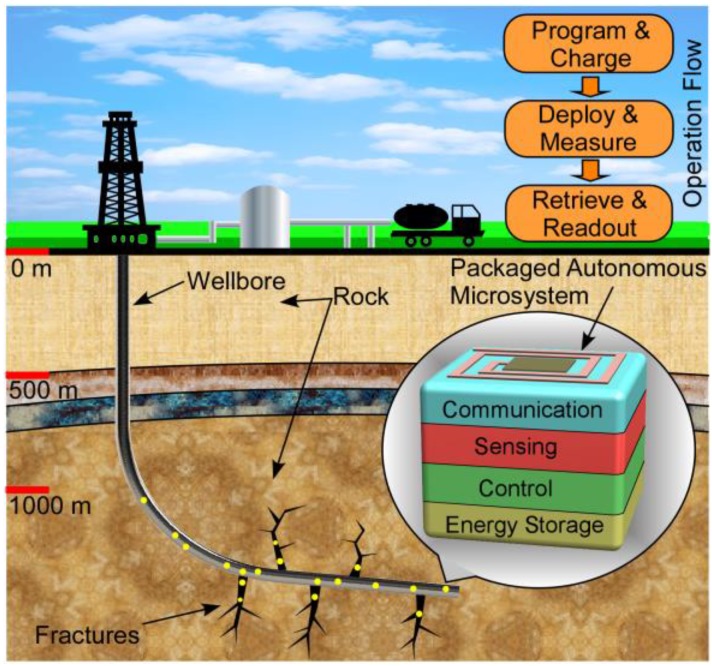
An example application scenario of the autonomous sensing microsystems in typical downhole environment.

It is envisioned that the traditional methods of illuminating the downhole environment can be augmented by the widespread deployment of autonomous sensing microsystems. Clusters of such microsystems can be used to enhance the spatial resolution of the sensed parameters, reaching locations in narrow confines and away from the wellbore. Figure 1 illustrates a potential application scenario in which the microsystems are sensing and logging data while being transported by fluid flow along the wellbore and the fractures. In the long term, the microsystems may be able to acquire time-stamped data on a variety of parameters, including temperature, pressure, chemical environment, geolocation, etc. At the end of the data collection period, the systems would be retrieved with the return fluid, and wirelessly interrogated at close proximity in controlled conditions [2].

At a depth of 12,000 feet (3658 m) in a typical reservoir, the temperature may reach 125 °C, the pressure may range from 1000 to 6000 psi (7–41 MPa), and the chemical environment may have salinity levels from 50,000 to 150,000 ppm [2]. In many other situations, the temperature and pressure may exceed 150 °C and 10,000 psi (69 MPa). Whereas the chemical and pressure tolerance can be provided by packages [6], the temperature tolerance must be built into the hardware. A consequence of the miniaturization of CMOS electronics is that the junction leakage current increases exponentially with temperature. For example, the leakage current of a memory circuit increases by 2000–10,000× when the temperature increases from room temperature to 125 °C [7,8,9,10]. High leakage current affects the performance of the electronics [11], and can lead to a substantial increase in power consumption, particularly in sleep mode. This increases the required battery capacity.

The operating temperature is a challenge for the battery as well. The uncertainty in the retrieval time mandates the use of rechargeable batteries; consequently, lithium batteries are attractive for these systems. Lithium batteries can provide high energy density and, in principle, can be used at temperatures as high as 250 °C [12,13,14]. However, there are very few options that are commercially available with mm-scale footprints, and none of these are rated at temperatures exceeding 100 °C. A Seiko MS412FE battery rated for 60 °C has a Φ4.8 mm footprint and 1 mAh capacity [15]. Thin film solid state lithium batteries can operate at temperatures up to 125 °C, and can be integrated on mm-scale silicon chips. However, this form factor compromises energy density; for example, the EnerChip CBC001-BDC thin film battery has only 1 µAh capacity on a 1.375 × 0.85 mm^2^ bare die [16].

Very limited work has been reported on microsystems for downhole environmental monitoring. Openfield™ Technology has developed a temperature and pressure recorder packaged in a Φ50.8 mm spherical titanium shell that can flow in well pipes for downhole monitoring [17]. Additionally, a research prototype using customized electronic chips encapsulated in a Φ7.5 mm polymer sphere has been reported for temperature and pressure measurements in oil wells [18,19].

**Figure 2:**
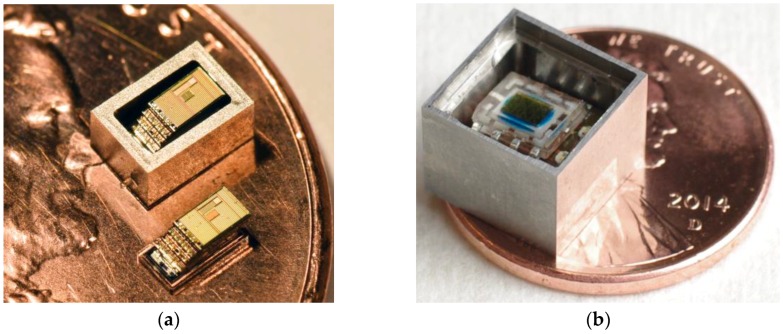
(**a**) Photo of the M^3^HT system stack (**bottom**) and inside a SS shell (**top**), on a US penny; (**b**) Photo of the ELM system in a SS shell before sealing with a sapphire lid, on a US penny.

In this paper, two autonomous sensing microsystem platforms are presented to demonstrate our efforts toward millimeter-scale systems for downhole environmental monitoring: the Michigan Micro Mote System for High Temperature (M^3^HT, Figure 2a), and the Environmental Logging Microsystem (ELM, Figure 2b). The M^3^HT uses customized electronic and battery chips to achieve an extremely small form factor and ultralow power consumption. The ELM system implements another approach to providing temperature tolerance that uses off-the-shelf electronic and battery components that can be assembled into a flexible printed circuit board (PCB). The preliminary versions of both systems are designed to record ambient temperature, which is stored during the deployment period, and reported by proximal wireless interrogation at the end of the deployment. Additional sensing capabilities will be incorporated in future generations. The designs and implementations of both systems are described in Section 2. Encapsulation of the system components in stainless steel (SS) or epoxy packages for the target environments is presented in Section 3. Experimental results of tests performed in high pressure, high temperature (HPHT) corrosive environments are described in Section 4, which is followed by discussion and conclusions in Section 5.

## 2. System Configuration

**Figure 3:**
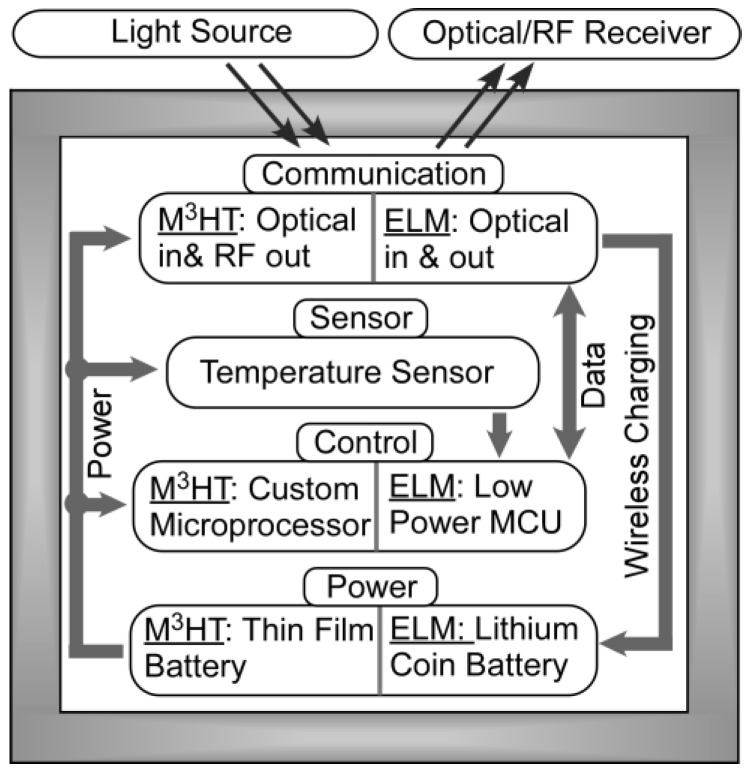
Functional block diagram of both the M^3^HT and ELM systems. Both systems consist of four modules: communication, sensor, control and power. Both systems are powered by a battery that is optically recharged.

The functional block diagram of the M^3^HT and ELM systems is shown in Figure 3. Each system includes a wireless communication module for setting operational parameters and reporting back data, a sensor module for measuring temperature, a control module for managing the system and processing and storing data, and a power module for supplying power to the system and recharging the battery. The design and performance parameters of both systems are summarized in Table 1. The implementation of each system is described below.

### 2.1. M^3^HT

The M^3^HT system is an enhancement of the previously reported Michigan Micro Mote (M^3^). The M^3^ system was intended for intraocular pressure monitoring; it had 2 × 1.5 mm^2^ footprint and <50 pW power consumption [20]. The system included an ARM Cortex M0 microprocessor, 3 kB memory, solar cell, power management module, 900 MHz radio communication module, and capacitive interface circuits for sensors. All circuit elements were customized to minimize energy consumption. Power was supplied by a Cymbet 1-µAh thin film lithium battery that could be charged by the solar cell.

The M^3^HT system uses a system configuration that is similar to the M^3^ system, with important changes that are necessary for operation at high temperatures. The primary challenge that must be addressed is the elevated leakage current in semiconductor junctions at high temperatures. The fabrication process used for M^3^HT is the conventional 180 nm CMOS technology from the Taiwan Semiconductor Manufacture Company (TSMC). The transistors in this technology are isolated by reverse-biased pn junctions, and the transistor leakage current at high temperatures is dominated by the reverse-biased pn junction leakage current. This current mainly consists of the diffusion current and the recombination-generation (R-G) current [21,22,23]. It can occur in two paths: from the substrate to the transistor wells, and from the wells to the source or drain of transistors. At elevated temperatures, the diffusion current dominates and is given by [21]:

(1) $$I_{Diffusion}=I_{S}[\exp(qV/kT)-1]$$

where *I_S_* is the reverse bias saturation current, *q* is the magnitude of the electronic charge, *V* is the reverse bias voltage, *k* is the Boltzmann constant, and *T* is the junction temperature in Kelvin. In this equation, *I_S_* has a mild dependence on temperature, while the primary temperature dependence comes from the exponential term. The consequence of the enhanced leakage is most significant for the memory circuit block; as the temperature is raised from 25 °C to 125 °C, the memory power consumption increases by ≈10,000× to ≈300 nW. For the implementation described in this work, the n-well biasing voltage is reduced from 1.2 V to 0.6 V, which results in 55% lower power consumption. The compromise is a reduction in immunity to power-supply noise, which is accommodated with the use of a large decoupling capacitor chip across the supply rails.

Another significant challenge is providing temperature immunity to the timer that is used to wake up the system each time a sensor measurement is to be taken. A temperature insensitive reference current generated by a bias circuit is used to stabilize the timer. This bias reference circuit achieves temperature independence by canceling the positive temperature dependency of the transistor subthreshold current with the negative temperature dependency of the voltage applied to the transistor gate [24]. With this improvement for the timer, the variation of the wake-up period over the temperature range of 25–125 °C is reduced from 80× to 1.4×.

An additional measure that is taken to accommodate the elevated power consumption at the target temperature is to enhance the power supply. This work uses two customized Cymbet lithium batteries, each with a capacity of 2 µAh, a nominal voltage of 4.1 V, and a footprint of 1.1 × 1.7 mm^2^. Rated by the supplier for operation at temperatures up to 70 °C, these rechargeable batteries lose capacity at higher temperatures. Specifically, we find that at 125 °C, the early discharge cycles are compromised by about 30% in capacity, and further reduced by another 50% after 6–7 discharge cycles. The internal resistance of the battery also significantly increases after the initial cycles. For scenarios where the sensing microsystems are deployed for 12–24 h per cycle and disposed after several cycles, this battery can provide an average current budget of 125–250 nA.

A customized on-chip electronic temperature sensor is used in the M^3^HT system [25]. This sensor consists of a temperature-sensitive oscillator, a reference oscillator, and digital counters. The oscillator-based sensing approach eliminates the need for an analog-to-digital converter (ADC), and allows the temperature sensor to consume as little as 4.6 nJ/conversion.

The M^3^HT system components are fabricated on 8 silicon chips, which include a processor chip, a radio chip, a sensor chip, a decoupling capacitor chip, a solar cell chip, an interface circuit chip to harvest energy from the solar cell, and two battery chips. These chips are stacked together and interconnected by wirebonds (Figure 2a). The overall chip stack is 2.9 × 1.1 × 1.5 mm^3^ (L × W × H) with a volume of 4.8 mm^3^. In principle, the height can be further reduced by thinning down each silicon chip.

### 2.2. ELM

An important distinction between the ELM and the M^3^HT is that the ELM uses packaged electronics to facilitate commercial assembly of the chips onto a commercial flexible PCB. The volume of the assembled system, therefore, is larger.

For this work, the C8051F990 low power microcontroller (MCU) from Silicon Laboratories, Inc. [26] was selected over other options. This MCU has an integrated temperature sensor, a small footprint of 3 × 3 mm^2^ and low power consumption (e.g., 80 µA in the active mode and 0.8 µA in the sleep mode) at room temperature. The linear voltage output of the temperature sensor is captured by the ADC embedded in the MCU, and the slope and offset of this output are calibrated and stored in the system software. The non-volatile flash memory embedded in the MCU is used for storing the program and measured data. This allows the data to be retained even after total depletion of the battery during deployment. In such a case, the ELM battery must be recharged following retrieval, prior to interrogation. Other MCUs that were also considered include the Freescale MPXY8300, the TI MSP430 series and the Microchip PIC12F1840. The ELM also includes an IXYS solar cell for charging the on-board rechargeable battery, and for receiving optical signals to set operational parameters. This solar cell provides a nominal output voltage of 4 V and short-circuit current of 50 µA in direct sunlight (6000 lux), which is adequate for recharging the battery [27]. Two light-emitting diodes (LEDs) are used to transmit data and system status information.

**Figure 4:**
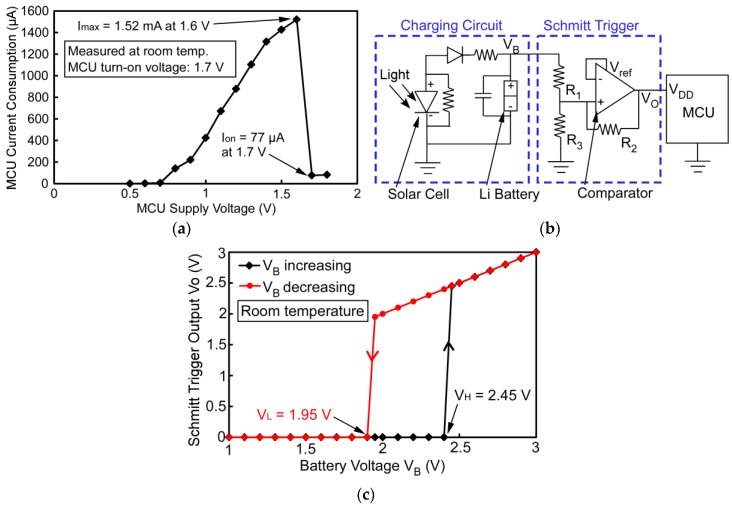
(**a**) Measured static current consumption of the C8051F990 MCU when the supply voltage is at or below the minimum operation voltage (1.8 V) specified in the MCU datasheet. A significant current spike, which peaks at 1.6 V supply voltage, is observed; (**b**) Schematic of the power supply circuit of ELM, including a charging circuit and a Schmitt trigger. The charging circuit includes a solar cell and other components to charge the Li battery. The Schmitt trigger serves as a power switch with hysteresis for the MCU; (**c**) Measured output of the Schmitt trigger (*V_O_*) while sweeping the battery voltage *V_B_*, showing hysteresis and the two threshold voltages, *V_H_* and *V_L_*.

A special power supply circuit is designed to manage low battery conditions that may be encountered in prolonged deployment periods. As shown in Figure 4a, the C8051F990 MCU consumes significantly higher current when its supply voltage drops below the minimum operation voltage (1.8 V) specified in the datasheet. In room temperature operation, the current draw reaches 1.52 mA at a supply voltage of 1.6 V, which is ≈19× the normal current draw in the active mode. This elevated current draw, which would be exacerbated at high temperatures, shortens the deployment lifetime. Additionally, it prevents the charging of depleted batteries before or after the deployment period, as this current draw significantly exceeds the combined maximum current available from the battery and the solar cell.

To address the problem of excess current draw by the MCU at low supply voltages, the ELM power supply circuit includes a Schmitt trigger along with the battery charging circuit (Figure 4b). The charging circuit uses the IXYS solar cell and other components to supply the necessary current for recharging the battery. The Schmitt trigger, implemented by a comparator (TI TLV3012) and three resistors, serves as a power switch with hysteresis to safely shut down the MCU before the supply voltage drops too low during deployment, and to prevent the MCU from turning on at lower battery voltages. As shown in Figure 4c, when the battery is being charged and its voltage (*V_B_*) is below the higher hysteresis threshold voltage (*V_H_*) of the Schmitt trigger, the Schmitt trigger is off and its output voltage (*V_O_*), which sets the supply voltage for the MCU, remains at 0. When *V_B_* reaches *V_H_*, the Schmitt trigger is turned on and *V_O_* follows *V_B_* closely; this also turns on the MCU. When the battery is discharging, the Schmitt trigger remains on until *V_B_* goes below the lower hysteresis threshold (*V_L_*), at which point the MCU is safely turned off. The values of *V_H_* and *V_L_* are determined by the three resistors [28]:

(2) $$V_{H}=\frac{1.2\times \left(R_{1}+R_{2}\|R_{3}\right)}{R_{2}\|R_{3}};\;V_{L}=\frac{1.2\times R_{3}}{R_{1}\|R_{2}+R_{3}}$$

For this work, *V_H_* is set to 2.45 V and *V_L_* to 1.95 V, allowing proper operation of the MCU during prolonged deployment periods, and effective battery recharging after deployment. The selected resistors are in the MΩ range to minimize the current consumption of the Schmitt trigger circuit.

The ELM system software is stored in the flash memory embedded within C8051F990 MCU. The software includes functions for all system operations, such as data recording and processing, power management, and optical communication. To save power, the software is designed so that the system stays in the sleep or idle mode whenever possible. One example is that during the wait time between measurements, the whole system is placed in sleep mode. Another example is that the MCU is placed in idle mode when the system is performing a task that takes an extended but predictable duration of time, e.g., when LEDs are flashed at a certain rate for data transmission.

The system clock oscillator must remain operational for the entire duration of all system operation modes. Its careful selection is essential for maintaining low power consumption. Three oscillators are embedded in the MCU: a low frequency oscillator (32 kHz) with low power consumption but high temperature dependency, and two high frequency oscillators (>20 MHz) that require large current draws but have negligible dependency on temperature. To maintain both low power operation and accurate timing in the system, the 32-kHz oscillator was selected, and its frequency is calibrated to one of the high frequency oscillators every time the system wakes up from sleep.

The power source selected for the ELM system was a lithium coin rechargeable battery MS412FE from Seiko Instruments [15]. This battery has a form factor of Φ4.8 mm × 1.2 mm, energy capacity of 1 mAh, and rated operating temperature of −20 °C to 60 °C. Our tests show that this battery, when fully charged at room temperature, can be subsequently deployed at 125 °C. In fact, it can support the ELM system in active measurement mode at 125 °C for at least two 6-h cycles without evident capacity loss, leakage, or any other sign of malfunction. After several initial cycles, the battery capacity degrades and the system can be discarded. The EnerChip CBC012 thin film battery from Cymbet™ Corporation was initially considered as a candidate, but was abandoned due to its limited battery capacity (12 µAh).

The flexible PCBs, fabricated and assembled by All Flex, LLC (Northfield, MN, USA), are folded in accordion fashion into three-fold stacks, with the solar cell and LEDs in the top layer, the battery sandwiched in the middle fold, and the MCU and other components on the bottom layer. The folded stacks are 6.5 × 6.3 × 4.5 mm^3^ (L × W × H) with a volume of 184.3 mm^3^. Approximately 50% of this volume is occupied by the surface mount packages of the electronic chips. As PCB assembly methods transition to chip-scale packaging [29], this is expected to decrease.

## 3. System Encapsulation

The systems are encapsulated in five-sided shells made from 17 to 4PH stainless steel, which provides impact and abrasion tolerance. The steel shells for the M^3^HT stack are 0.35 mm thick (Figure 2a), and are filled with a proprietary black epoxy by RTI International that is designed to withstand elevated temperatures. The opaque nature of this epoxy is beneficial in minimizing photo-induced stray current when the electronics are operated in ambient light. However, the top surface of the stack, where the solar cell is located, is covered by transparent epoxy to allow optical charging. The encapsulated M^3^HT systems made by this approach are 4.2 × 2.2 × 2.8 mm^3^ in size. To enhance the RF communication range, additional M^3^HT systems are encapsulated in epoxy without the steel shells, which could otherwise shield the antenna.

For the ELM systems, the shells are capped with sapphire lids instead of being filled with epoxy (Figure 2b). The steel walls and the sapphire lid are all 1 mm thick. The transparency of the sapphire lid is necessary to charge the battery and to allow optical communication. The seal between the shell and the lid is formed using Masterbond^®^ epoxy EP42HT-2. An anti-corrosion coating of Parylene^®^ is deposited on all the outer surfaces of the packages. This method is known to produce a hermetic seal that can withstand the intended test conditions [6]. The encapsulated ELM systems are 8.9 × 8.9 × 6.9 mm^3^ in size.

## 4. Test Results

**Figure 5:**
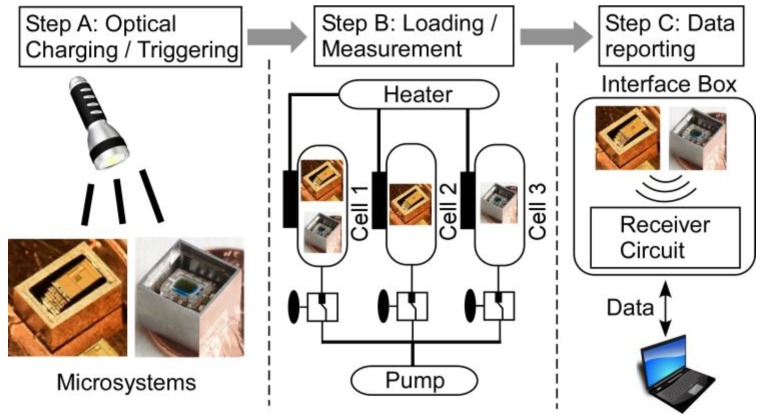
The testing procedure diagram. In Step A, the systems were charged and triggered into the detection state. In Step B, the systems were inserted into the testing cells with HPHT conditions. In Step C, the measured temperature data was reported wirelessly to a laptop through an interface unit.

The encapsulated M^3^HT and ELM systems were subjected to a series of HPHT tests in liquid ambients that emulated certain downhole conditions (Figure 5). The tests were performed in three steps. In Step A, the systems were fully charged using a light source and then triggered into their respective detection states, in which temperature readings were taken at predefined time intervals. The systems were then sealed into plastic bags, each of which was filled with one of the three selected test liquid media: American Petroleum Institute standard brine (API brine, CaCl_2_ 2 wt % and NaCl 8 wt % in deionized H_2_O); Isopar™-L (synthetic isoparaffinic hydrocarbon fluid from Exxon Mobil); and uncured cement slurry. The bags were made from a high-grade nylon 6 film that can tolerate the target temperatures. In Step B, the plastic bags, with the systems and test solution inside, were inserted into HPHT testing cells that were filled with mineral oil. Following this, the cells were elevated to the target pressure. The temperature was then gradually ramped up for ≈30 min. After the prescribed soak time, the pressure was released and the temperature was allowed to cool gradually for ≈30 min. In Step C, the systems were retrieved from the cells, cleaned, and interrogated by RF or optical means as appropriate.

**Figure 6:**
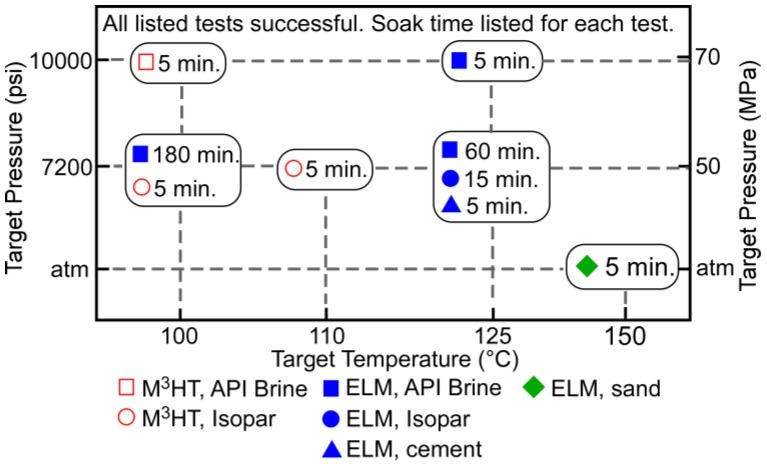
Summary of HPHT test results. All tests listed in the figure were successful. The soak time for each test is shown. The total time for each test included the soak time, ≈30 min ramp up, and ≈30 min ramp down.

The HPHT test results are summarized in Figure 6. Freshly charged M^3^HT systems were used in each test. One test was performed at 100 °C temperature and 7200 psi pressure in Isopar-L in order to validate the customized electronics and system encapsulation at the elevated temperature and pressure. The soak time for this test was 5 min, making the total duration of the test ≈65 min. The test was successful; temperature data was recorded approximately every minute during the test and was reported upon interrogation, and both types of system encapsulation used for the M^3^HT survived the test. Another test was performed with similar conditions, but at 110 °C to evaluate the electronics at the higher temperature; this test was also successful. In another test, M^3^HT systems with full epoxy encapsulation successfully survived higher pressure of 10,000 psi at 100 °C temperature in API brine for 5 min soak (i.e., 65 min total duration). The system with steel shell was not deployed in this test.

Freshly charged ELM systems were evaluated at 125 °C temperature and 7200 psi pressure applied separately in environments of API brine for 60 min soak, Isopar-L for 15 min soak, and cement slurry for 5 min soak, in order to evaluate the electronics and encapsulation in these conditions. The total durations of these tests were ≈120 min, ≈75 min, and ≈65 min, respectively. Temperature data was recorded every 2 min during the tests. The ELM systems passed each of these tests. Another test was performed at 125 °C temperature and 10,000 psi pressure in API brine for 5 min soak (i.e., ≈65 min total duration) to evaluate the system encapsulation under the higher pressure, and the systems survived. Another test was performed for a 3-h soak (4 h total duration) at 100 °C and 7200 psi pressure in API brine to evaluate extended deployment; this test was also successful.

In summary, both types of systems passed all tests that are identified in Figure 6. Measured temperature data were successfully reported following each test. The ELM systems survived the HPHT conditions at up to 125 °C and up to 10,000 psi. The M^3^HT systems survived at a slightly reduced temperature of 110 °C and pressure of 7200 psi, and separately at 100 °C and 10,000 psi. Both types of systems survived all three test solutions: API brine, Isopar-L, and cement slurry. The ELM systems also survived longer tests with soak times of 1 h at 125 °C and 7200 psi, and 3 h at 100 °C and 7200 psi.

Separately, the ELM systems were tested at up to 150 °C at atmospheric pressure (Figure 6). For these tests, the systems were immersed in dry sand to provide temperature uniformity. The systems successfully performed the temperature logging function and reported data upon interrogation.

## 5. Discussion and Conclusions

The encapsulated M^3^HT and ELM systems were successfully tested in HPHT conditions in corrosive ambient environments. This suggests that both systems can potentially survive certain downhole environment with similarly harsh conditions. Specifically, the successful tests indicate that the challenges in the M^3^HT electronics were adequately addressed for operation at temperatures at least up to 110 °C. The electronic and software configurations used in the ELM allowed operation at temperatures reaching 150 °C despite the use of standard commercial components that are rated only to 85 °C. The selected batteries for both systems could support system operation at these high temperatures. The system packages were shown to provide adequate protection from the high pressure and corrosive conditions for at least three hours.

The electronic chips for both systems were fabricated using standard CMOS technology that is not optimized for high temperature operation. Specialized electronics for temperatures well above 200 °C use technologies such as trench isolation, silicon-on-insulator (SOI) substrates, and other variations of the standard CMOS process [7]. Among these, the trench isolation approach uses enhanced isolation between adjacent transistors, whereas the SOI substrate approach uses an insulation oxide layer buried beneath the transistor layer to reduce the leakage current from the source or drain of the transistor to the substrate. Silicon carbide electronics also have excellent high temperature performance, but will serve as an option only after the scale of integration has been significantly advanced [30].

Rechargeable batteries at the millimeter scale that also provide enduring high-temperature operation remain a challenge. The thin film rechargeable lithium battery used in M^3^HT was based on the chemistry of lithium phosphorous oxynitride (LiPON) [31]. While it offers an aggressive form factor, it is limited in energy density and battery capacity (to a few µAh or less), and consequently limits deployment lifetime. The commercially available coin battery used in ELM uses manganese silicon lithium [15]. It has high energy density, but its miniaturization is limited by its package. It has a rated temperature range up to 60 °C, and its performance is degraded significantly at higher temperatures. Batteries based on lithium thionylchloride (LiSOCl_2_) become appealing for oil field applications due to outstanding performance at high temperature [12]. Non-rechargeable batteries based on this chemistry have been developed by Tadiran Batteries for operation at 150 °C. However, the smallest available option has a cylinder shape with a size of Φ24.5 mm × 5.8 mm [32]. Even though this chemistry is excellent at high temperature, the inability to recharge is a problem for the intended application. Batteries with smaller form factor have not been made available yet for this chemistry.

Harvesting of mechanical, thermal or chemical energy in the downhole environments may not be a viable solution as a sole power source, due to uncertainty in available power level and its continuity. However, in the long term it may be possible to incorporate this along with a rechargeable battery to supplement the limited battery capacity.

In conclusion, this work has demonstrated that autonomous microsystems can be made for downhole environments for operating temperatures at least as high as 110–150 °C using conventional junction-isolated CMOS processes and lithium-based batteries. Microsystems that use customized circuit design methods, thin-film batteries, and a wirebonded stack arrangement can be implemented in a volume of 2.9 × 1.1 × 1.5 mm^3^. Microsystems that use commercial off-the-shelf electronics, coin cell batteries, and flexible PCB substrates, can be designed to mitigate certain temperature effects; these microsystems can be implemented in a volume of 6.5 × 6.3 × 4.5 mm^3^. The larger volume of such systems is consumed by larger batteries that are necessary to accommodate the higher current draw and the surface mount chip packages that are necessary to facilitate the commercial PCB assembly. As shown, both types of microsystems can be hermetically encapsulated to tolerate corrosive and high-pressure environments. At elevated temperatures, the chemistry of commercially available miniature batteries constrains the active data collection period—typically to several hours. High-temperature batteries will greatly extend the active deployment lifetimes.

Looking forward, additional sensing modalities are of high interest for integration into either of the two microsystem platforms, including pressure, acoustic, inertial, and chemical sensors. The integration with these sensors is very promising and currently underway.

## Figures and Tables

**Table 1:** Design and performance parameters of the M^3^HT and ELM systems.

Parameter	M^3^HT System	ELM System
Stack size (L × W × H)	2.9 × 1.1 × 1.5 mm^3^	6.5 × 6.3 × 4.5 mm^3^
Package size (L × W × H)	4.2 × 2.2 × 2.8 mm^3^	8.9 × 8.9 × 6.85 mm^3^
Integration method	Wire bonded stack	Flexible PCB
Packaging materials	SS shell filled with epoxy	SS shell with sapphire lid
Nominal operating voltage	4.1 V (converted to 1.2 V/0.6 V)	3.0 V
Power consumpt. (sleep, 125 °C)	2.7 µW	30 µW
Operating frequency	0.2 Hz sleep/740 kHz active	32.768 kHz sleep/active
Wireless comm. method	RF 900 MHz/Optical	Optical
Battery type: rechargeable Li	Thin film, custom	Coin cell, commercial
Battery size	1.7 × 1.1 × 0.15 mm^3^ each, ×2	Φ4.8 mm × 1.2 mm
Battery capacity	2 µAh each, ×2	1 mAh
Optical recharging	Yes	Yes
